# Occupational Safety & Health Management and Corporate Sustainability: The Mediating Role of Affective Commitment

**DOI:** 10.1016/j.shaw.2023.10.006

**Published:** 2023-10-12

**Authors:** Zhen Chao Tan, Chun Eng Tan, Yuen Onn Choong

**Affiliations:** 1 Department of Economics and Corporate Administration, Faculty of Accountancy, Finance and Business, Tunku Abdul Rahman University of Management and Technology, Malaysia; 2 Department of Business and Public Administration, Faculty of Business and Finance, Universiti Tunku Abdul Rahman, Malaysia

**Keywords:** construction companies, corporate sustainability, employee affective commitment, occupational safety & health management

## Abstract

**Background:**

Occupational safety & health management (OSH) has garnered greater attention for its significance in promoting corporate sustainability for organizations in recent decades. The construction industry, in particular, is a major contributor to Malaysia's thirst for corporate sustainability in order to provide long-term support for the country. Thus, the main tenet of this study is to examine the mediating effect of employee affective commitment on the relationship between OSH and corporate sustainability.

**Methods:**

A questionnaire was administered to 273 full-time employees of listed construction companies in Malaysia. Smart PLS software version 3 was used to test the proposed model and hypotheses. Both the measurement model and the structural model were evaluated.

**Results:**

According to the findings, OSH and its dimensions are positively related to employee affective commitment. Employee affective commitment, on the other hand, has been found to be significantly related to corporate sustainability and its dimensions: economic, social, and environmental sustainability. Apart from this, the prominent results reveal that employee affective commitment partially mediates the relationship between OSH and corporate sustainability and its dimensions: economic, social, and environmental sustainability.

**Conclusion:**

This empirical finding adds to the existing literature in explaining how OSH and affective commitment led to corporate sustainability. Several implications are offered to various stakeholders, such as construction companies, policymakers, and relevant regulators.

## 1 Introduction

Throughout the years, sustainability has become a business imperative and a critical practice for organizations. There has been an increase in corporate commitments to lead and promote myriad types of sustainability [1]. Corporate sustainability is defined as an organization's ability to meet the direct and indirect needs of stakeholders continuously, while ensuring its long-term viability [2]. According to Dumitriu et al. [3], it is important for organizations to achieve sustainability as it can enhance their public image, brand awareness, cost savings, and numerous other benefits. Ultimately, it creates competitive advantages for an organization and makes it more sustainable than its competitors. It is undeniable that Mala-ysian organizations are also pursuing these benefits by striving to achieve their sustainability goals [4].

In view of this, the individuals behind the scenes, such as the employees of an organization, play a crucial role in driving corporate sustainability [5]. Nurman et al. [6] have postulated that human beings are central to sustainable development. Thus, employees are one of the primary factors contributing to a company's ability to achieve competitiveness. However, workplace accidents present a significant challenge for organizations, impeding their progress towards corporate sustainability [7], as they directly jeopardize the well-being of the workforce. According to the International Labor Organization [8], occupational accidents rank as the third leading cause of death globally. Annually, there are approximately 340 million occupational accidents and 160 million work-related illnesses reported. Furthermore, an estimated 2.3 million individuals worldwide lose their lives due to work-related accidents or diseases each year. In addition, approximately 340 million occupational accidents occur annually. In Malaysia, the number of occupational accidents in the construction sector has experienced a significant increase of 200%, rising from 3,345 cases in 2015 to 7,984 cases in 2019 [9]. However, there has been a decline in the number of occupational accidents between 2020 and 2021, with 3,958 cases in 2020 and 2,297 cases in 2021. One of the primary factors contributing to the decrease in the number of occupational accidents was the government-imposed lockdown to control the spread of COVID-19. Conversely, the fatality rate has shown an increase from 10.7 to 12.8, resulting in a 20% rise.

There is no doubt that financial profitability is one of the key factors driving the success of an organization. However, relying solely on this perception is not sustainable, as an organization solely focused on profit may end up exploiting its employees without making any value-added efforts. People have overlooked the significance of employees in contributing to the creation of value for shareholders and stakeholders, including consumers. If employers persist in prioritizing profit over investing in their employees, such as providing a safe and healthy workplace, employees may become demotivated and disengage from the organization [10], resulting in higher rates of workplace accidents. In light of this, employers must take necessary steps to manage occupational safety and health (OSH) at the workplace to ensure the ongoing well-being of their employees and their commitment to the organization. Implementing proactive OSH management practices can effectively reduce workplace accident rates. This, in turn, fosters employee commitment and contributes to the overall sustainability of the organization [11,12].

This paper has two main objectives. Firstly, we aim to conduct a rigorous examination by testing the dimensionality of OSH management in relation to affective commitment. While previous studies have often considered and tested OSH as a whole, our study intends to fill this gap by examining the effects of each dimension of OSH on employees' affective commitment [[13], [14], [15]]. This approach allows employers to identify specific dimensions in which their organization may be lacking and enables them to target those areas for improvement in order to enhance workplace safety, which in turn affects employee behavior. It is important to recognize that a lack of control over workplace safety and health can have negative consequences on employees' emotions, commitment, and performance [16]. Restoring employees' commitment to the organization can reduce the costs associated with injuries and enhance overall employee performance. Ultimately, this contributes to the long-term corporate sustainability of the business.

The second focal point of this paper is to investigate the mediating effect of employee affective commitment on the relationship between perceived sound OSH management and corporate sustainability. Previous research has primarily focused on examining the impact of employees' behavior on corporate sustainability [[17], [18], [19], [20], [21]]. However, limited attention has been given to the influence of employees' perceptions of OSH management in predicting corporate sustainability through employee affective commitment [22,23]. We claim that when management or the OSH committee effectively addresses safety, health, and welfare concerns, it tends to increase employees' commitment, thereby enhancing company performance and achieving corporate sustainability. Conversely, we argue that if employees do not feel a sense of safety and health in the workplace, they will not form an emotional attachment to the company and may not fully commit to their job, perceiving the company as merely a workplace rather than a second home [24]. This lack of emotional attachment has a negative impact on company performance and hinders the achievement of long-term corporate sustainability [25].

This study is expected to make two important contributions. Firstly, by examining the dimensions of OSH management on corporate sustainability, this study contributes to the growing body of knowledge on OSH management and corporate sustainability. Secondly, this study develops a comprehensive research model by incorporating employee affective commitment as a mediator. This model provides practical and theoretical insights to various stakeholders, including practitioners, researchers, individual construction companies, and relevant government agencies.

### 1.1 Proposed research model and hypotheses development

According to Fredrickson [26], the broaden-and-build theory (BBT) focuses on the influence of positive emotions (e.g., joy, interest, contentment, pride, love) and negative emotions (e.g., sadness, fear, anger) in the process of human adaptation. Positive emotions have the capacity to broaden thought-action repertoires and foster the development of personal resources [26,27]. This implies that cultivating positive emotions can enhance individuals' creative thinking, understanding, and behavior. BBT elucidates how positive emotions can aid in expanding an individual's capacity to cope with negative emotions.

Based on BBT, it can be inferred that employees' perception of OSH management, which triggers positive emotions in the workplace, leads to cognitive broadening. For example, when employees perceive beneficial actions taken by their company, such as improving safety measures or implementing a safety program, they are more likely to experience positive emotions. Consequently, this contributes to the development of personal resources [26,27]. In light of this, employees who have previously encountered such positive experiences are likely to exhibit attitudinal and affective reactions towards their job, as well as a sense of identity within the organization. Job satisfaction refers to an individual's attitude or affective response to their job [28], while organizational commitment pertains to an individual's sense of belonging to the organization [29]. This aligns with a study conducted by Vacharkulk-semsuk and Fredrickson [30], which suggests that individuals who experience happiness not only have more positive emotions but also develop valuable psychological resources that contribute to their well-being and overall satisfaction.

Affective commitment is defined as an employee's perceived emotional attachment to their organization. Affective commitment mainly “captures the emotional identification, engagement, and attachment that an individual has towards his or her organization” [31]. Based on the BBT of positive emotions, affective commitment can be treated as a behavioral phenomenon that results from effective OSH management. OSH management is defined as the processes concerned with ensuring the safety, health, and well-being of people engaged in work or employment [32]. OSH is a holistic approach to the total well-being of people at work or in employment. For instance, organizational management actively ensures employee safety and health in the workplace, which includes providing personal protection and safety equipment, continuous updating of OSH guidelines, the opportunity to attend safety and health programs, and frequent on-site inspections of employee compliance. If employees perceived a greater sense of safety and health with the support of management, they would feel happy and comfortable staying with the organization, which subsequently induces them to work energetically and results in greater affective commitment [33,34].

With positive affective commitment, employees feel more loyal, share organizational values, are committed to their jobs, and willing to perform and act according to the organization's vision and mission [35]. Ha-Brookshine [36] stated that employees' commitment is one of the key successes for corporate sustainability. Lee [37] further argued that employees' commitment plays an essential role in directly and indirectly influencing an organization's corporate sustainability performance. Huan et al. [17] found that there is a positive relationship between organizational commitment and economic sustainability. Employees who have a high commitment towards their organization tend to improve the organization's performance (i.e., revenue and productivity), and such performance is one of the most crucial antecedents in determining the organization's economic sustainability. Likewise, human capital elements must be effectively leveraged in accordance with the organization's sustainability principles to maintain its sustainability [38]. Patiar and Wang [26] also postulated that organizational commitment is positively related to socially sustainable performance (i.e., support for the local community), whereby employees who commit to their organization tend to assist the organization in achieving social sustainability. Employees who are committed to their organization would be more likely to provide help to their co-workers at work and thus foster good social sustainability practice. Lastly, environmental factors must also be taken into consideration by an organization to sustain its business activities and performance. There is a positive relationship between organizational commitment and environmental sustainability, as shown in the study by Pinzone et al. [18]. They further argued that employees who are committed to the environmental goals of their organization tend to pursue the green values of the organization. According to the BBT, if employees have an emotional attachment to their organization, it broadens their awareness, contentment, and love, which encourages them to produce positive actions that contribute to organizational performance for corporate sustainability [39,40].

Kundu and Gahlawat [41] further justified the idea that a safe working environment provides employees with a sense of security and motivation in their job. A positive organizational environment motivates both individuals and organizations, and this effect is important in fostering innovation for long-term sustainability [42]. Therefore, the adoption of BBT in this study is appropriate to explain the indirect effect of employees' perceptions of OSH management on corporate sustainability through the mediation of organizational commitment. In other words, it is proposed that OSH could activate employees' positive affect (the psychological resources that are built into employees), whereas BBT can broaden employees' actions by making them work harder and become emotionally attached to their organization. This further contributes to performance at the individual, team, and organizational levels, which results in corporate sustainability. Kaynak et al. [43] revealed that employees who value their organization's OSH management effort in ensuring their safety, health, and welfare would enhance their commitment towards the organization. The OSH management that engages in quality OSH management practices as a measure to ensure employees' safety and health in the workplace would benefit because their employees would reciprocate with various forms of positive behavior such as affective commitment, which in turn contributes to corporate sustainability performance [37].

**Fig. 1:**
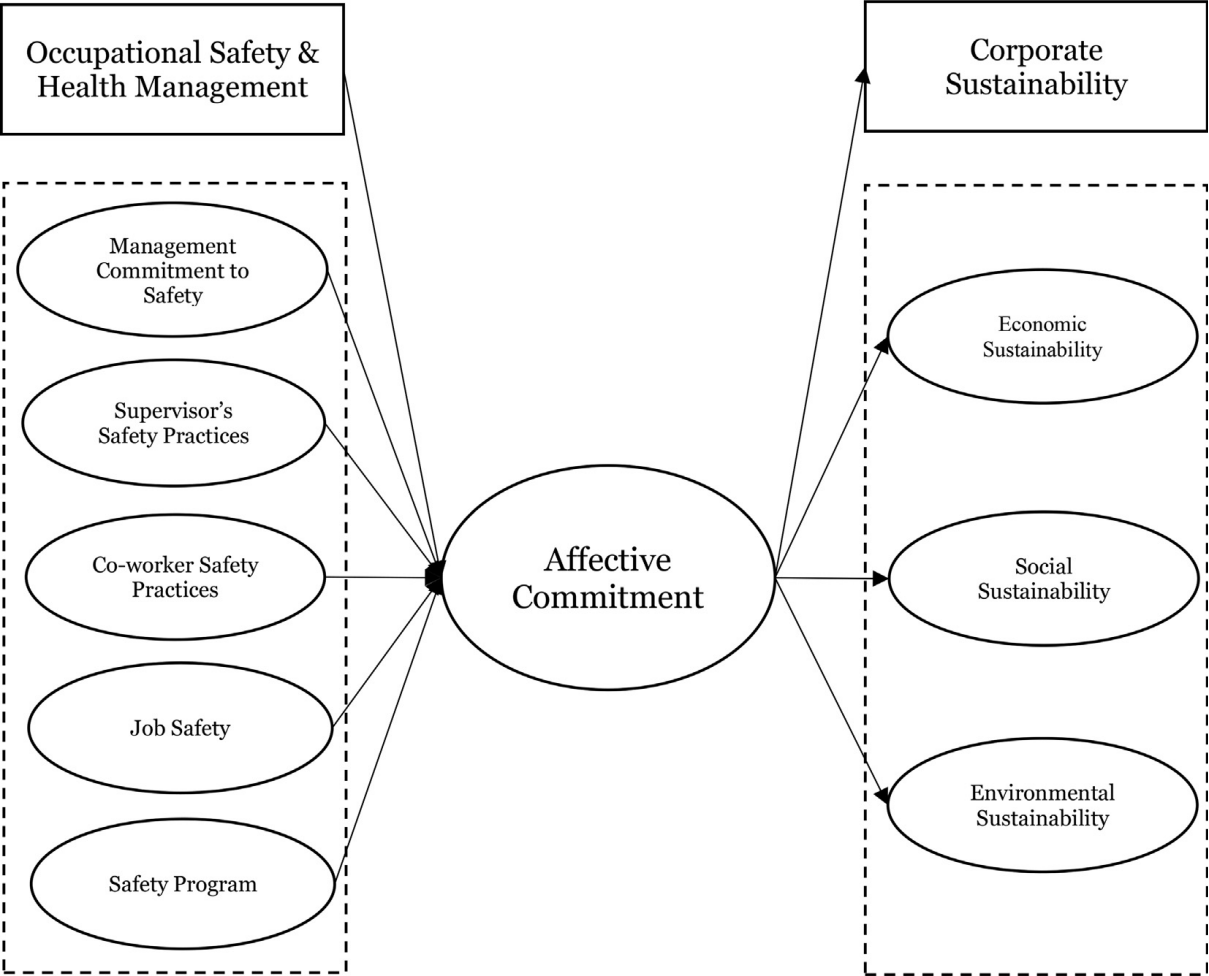
The proposed research model.

In view of the discussion, a research model is proposed (Fig. 1). With the support of 10.13039/501100010471BBT, it is argued that affective commitment could serve as a mediating mechanism in the relationship between occupational safety & health management and corporate sustain-ability.

Hence, we proposed several hypotheses as follows.

H1 Occupational safety & health management is positively related to employee affective commitment.

H1 (a-e): (a) Management commitment to safety, (b) supervisor's safety practices, (c) co-worker safety practices, (d) job safety, and (e) safety program is positively related to affective commitment.

H2 Affective commitment is positively related to corporate sustainability.

H2 (a-c): Affective commitment is positively related to (a) economic sustainability, (b) social sustainability, and (c) environmental sustainability.

H3 The relationship between occupational safety & health management and corporate sustainability is mediated by employee affective commitment.

H3 (a-c): The relationship between occupational safety & health management and (a) economic sustainability, (b) social sustainability, and (c) environmental sustainability is mediated by affective commitment.

## 2 Materials and methods

### 2.1 Procedures and samples

The data was collected using a self-administered survey on public-listed construction companies in Malaysia. A standardized questionnaire was distributed along with a cover letter containing the purpose of the study. A total of 273 respondents were recruited through a convenience sampling technique. All participation was voluntary and anonymous. There were two selection criteria: (1) they had to be full-time employees and (2) they had to work for Malaysian-listed construction companies. Prior to the data collection, permission for data collection was applied for and granted by the human resources department of the respective construction companies. Each targeted respondent was given one week to complete and return the questionnaire directly to the researchers with the postage stamps and envelope provided. A total of 500 questionnaires were distributed to potential respondents, with only 273 usable questionnaires returned, giving a response rate of 54.6%. The sample was comprised of 256 (94%) male respondents and 17 (6%) female respondents. The majority of the respondents fall within the age group of 38-47 (37%, n = 101) and the tenure duration of 5-6 years (31%, n = 84).

### 2.2 Research instruments

Occupational safety & health management consists of five higher-order dimensions with a 25-item measurement scale. Chen et al. [44] and Lu and Shang [45] identified six items for management commitment to safety, six items for supervisors' safety practices, four items for co-worker's safety practices, four items for job safety, and five items for a safety program. Management commitment to safety is explained as the extent to which the management and safety personnel are perceived to impose a high priority on safety, communicate, and react effectively to safety issues [46]. Sample item includes “My management provides an adequate safety training program.” The supervisor's safety practices are described as the supervisor's commitment in their direct role (e.g., keeping track of unsafe practices, acknowledging workers who adopt safe work behaviors, communicating safety policies, etc.) in practicing safety [47]. Sample item includes “My supervisor spends time showing me the safest way to do things at work.” Chen et al. [44] defined co-worker safety practices as the worker's perception about whether their co-workers have good safety behaviors, such as wearing and encouraging co-workers to wear personal protective equipment. The sample item is “My co-workers ignore safety rules.” Job safety is defined as workers' perceptions of the safety principles, values, norms, beliefs, and practices of other workers in their work environment [48]. The sample item is “The work at the site is unsafe.” Finally, a safety program can be referred to as the practices or activities that are undertaken by the organization to reduce unsafe conditions and acts in the workplace [47]. The sample item includes “The design of the safety training program is good.” All items were used to examine the perceptions of various stakeholders' initiatives, including management, supervisors, and co-workers, such as safety measures taken in the workplace and safety training.

Affective commitment was measured using eight items developed by Allen et al. [49]. A sample item on this scale includes “I would be very happy to spend the rest of my career with this organization.” These items were adopted to examine employees’ perceived emotional attachment towards their organization.

Corporate sustainability is comprised of three higher-order dimensions with a 16-item measurement scale by Martinez et al. [50] and Sarango-Lalangui et al. [51]: four items for economic sustainability, six items for social sustainability, and six items for environmental sustainability. Sample items include “My organization obtains the greatest possible profits” for economic sustainability, “My organization helps to solve social problems” for social sustainability, and “My organization cares for and protects the environment” for environmental sustainability. This 16-item scale reflects the employee's perception of their employer's or corporation's ability to pursue long-term stakeholder value through effective business strategies by focusing on economic, social, and environmental aspects of doing business.

All adapted measurement items were measured on a seven-point Likert scale ranging from 1 (strongly disagree) to 7 (strongly agree). Some items were modified to suit the context of the current study. The full list of items can be referred to Appendix A. The questionnaire was also translated into Malay and Chinese by using back-to-back translation method [52]. These measurement items and the questionnaire were pre-tested and pilot-tested with 30 respondents to ensure the relevancy of the items in the construction sector context. Thus, this has confirmed the face validity.

### 2.3 Data analysis

Two software packages, Statistical Package for the Social Sciences version 25 and Smart PLS version 3.2, were deployed for data analysis. At first, Statistical Package for the Social Sciences software was pursued to conduct descriptive analysis: mean, standard deviation, scale reliability, common method variance, and inter-correlation analysis. Whereas, for Smart PLS, it was used to assess the measurement model and structural model. The two-stage analytical approach was followed [53] to examine and report the validity result, reliability result, significance of the predicted paths with t-statistics, and explanatory power of the proposed model.

### 2.4 Descriptive analysis

All variables were subjected to descriptive statistics, inter-item reliability scales, one-factor tests, and correlations. Table 1 shows the internal consistency score for all higherorder constructs, and lower-order constructs were well above 0.700 as specified by Hair et al. [54]. The Harman single factor test was examined and found that none of the factors was able to explain 50% of the variances in corporate sustainability [55]. Thus, the common method bias is not a serious threat in this set of data. For inter-correlations, it shows that all constructs are significantly correlated with each other (see Table 1).

**Table 1:** Mean, standard deviation and inter-correlations

No.	Construct	1	2	3	4	5	6	7	8	9	10	11	12
1	Age												
2	Tenure	.45∗∗											
3	Firm Size	−.13∗	−.08										
4	MCS	.02	.02	.10									
5	SSP	−.08	.05	.17∗∗	.11								
6	CSP	−.01	.09	.16	.09	.69∗∗							
7	JS	−.01	.19∗	.03	.09	.60∗∗	.68∗∗						
8	SP	−.01	.08	.02	.11	.75∗∗	.62∗∗	.64∗∗					
9	EAC	.00	.10	.07	.17∗	.79∗∗	.69∗∗	.69∗∗	.81∗∗				
10	EcS	−.05	.06	.10	.09	.65∗∗	.60∗∗	.55∗∗	.69∗∗	.75∗∗			
11	SoS	−.02	.05	.14	.13	.76∗∗	.70∗∗	.55∗∗	.76∗∗	.74∗∗	.69∗∗		
12	EnS	−.09	.07	.10	−.04	.63∗∗	.60∗∗	.49∗∗	.73∗∗	.65∗∗	.67∗∗	.70∗∗	
	Mean	N/A	N/A	N/A	5.24	5.48	5.52	5.39	5.40	5.48	5.41	5.60	5.41
	SD	N/A	N/A	N/A	0.88	0.67	0.75	0.76	0.61	0.61	0.65	0.63	0.64
	CA	N/A	N/A	N/A	0.93	0.85	0.81	0.81	0.81	0.87	0.81	0.86	0.83

*Note.* ∗, significant at 0.05; ∗∗, significant at 0.001; CA, Cronbach Alpha; CSP, Co-worker Safety Practices; EAC, Employee Affective Commitment; EcS, Economic Sustainability; EnS, Environmental Sustainability; JS, Job Safety; MCS, Management Commitment to Safety; SP, Safety Program; SSP, Safety Supervisor's Practices; SoS, Social Sustainability; SD, Standard Deviation.

### 2.5 Measurement model

As all measurement items were adopted from past empirical studies, all items were validated by performing construct validity tests. Both convergent validity and discriminant validity were tested and confirmed with the Smart PLS algorithm technique. Table 2 demonstrates that the average variance extracted for constructs exceeds the threshold value of 0.500 [54], that ranges from 0.530 to 0.782. This shows that at least 50% of the variance of each construct is explained by its measurement items [56]. Except for two items from employee affective commitment, three items from co-worker safety practices, one item from supervisor's safety practices, and two items from environmental sustainability, all items were with loadings of 0.708. These eight items with indicator loadings fall between 0.600 and 0.708. Thus, none of the items are discarded from the measurement model if the average variance extracted score for its construct surpasses the 0.500 cut-off value [54]. For composite reliability, the score for constructs ranged from 0.861 to 0.937, which is well above the minimum score of 0.700 [54]. Therefore, it is affirmed that the items of each construct have revealed adequate convergent validity.

**Table 2:** Convergent validity result

Constructs	AVE	CA
Management’s commitment to safety	0.722	0.939
Supervisor's safety practices	0.578	0.891
Co-worker safety practices	0.630	0.872
Job safety	0.637	0.875
Safety programme	0.561	0.865
Occupational safety & health management	0.604	0.873
Employee affective commitment	0.523	0.897
Economic sustainability	0.634	0.874
Social sustainability	0.593	0.897
Environmental sustainability	0.540	0.875
Corporate sustainability	0.790	0.918

Note. AVE, Average Variance Extracted; CA, Composite Reliability.

Table 3 depicts the Heterotrait-Monotrait criterion (HTMT) scores for the assessment of discriminant validity. It is confirmed that none of the correlation values is greater than the threshold value of the HTMT_0.90_ criterion [54]. Thus, the measurement model illustrated sufficient discriminant validity.

**Table 3:** Heterotrait-Monotrait ratio

No	Construct	1	2	3	4	5	6	7	8	9
1	MCS									
2	SSP	0.146								
3	CSP	0.109	0.818							
4	JS	0.099	0.715	0.833						
5	SP	0.120	0.674	0.766	0.783					
6	EAC	0.193	0.723	0.822	0.816	0.845				
7	EcS	0.098	0.784	0.735	0.669	0.853	0.811			
8	SoS	0.151	0.879	0.833	0.654	0.781	0.864	0.818		
9	EnS	0.044	0.736	0.739	0.592	0.897	0.771	0.822	0.823	

*Note.* CSP, Co-worker Safety Practices; EAC, Employee Affective Commitment; EcS, Economic Sustainability; EnS, Environmental Sustainability; JS, Job Safety; MCS, Management Commitment to Safety; SSP, Safety Supervisor's Practices; SP, Safety Program; SoS, Social Sustainability.

## 3 Results

As directional hypotheses were formulated, a one-tail test was pursued by conducting the bootstrapping technique with 2,000 resamples. The explanatory power for employee affective commitment (R2 = 0.761) is considered substantial, indicating that the five dimensions of OSH in this study can explain at least 76.1% of the variance in employee affective commitment. On the other hand, the explanatory power for corporate sustainability (R2 = 0.749) is considered moderate, suggesting that 74.9% of the variance in corporate sustainability can be explained by employee affective commitment. Therefore, we can conclude that both OSH and empl-oyee affective commitment have a strong influence on the outcome variables of affective commitment and corporate sustainability. Moreover, the in-sample predictive power score does not exceed the value of 0.90, which indicates that the model fits the data [54].

**Fig. 2:**
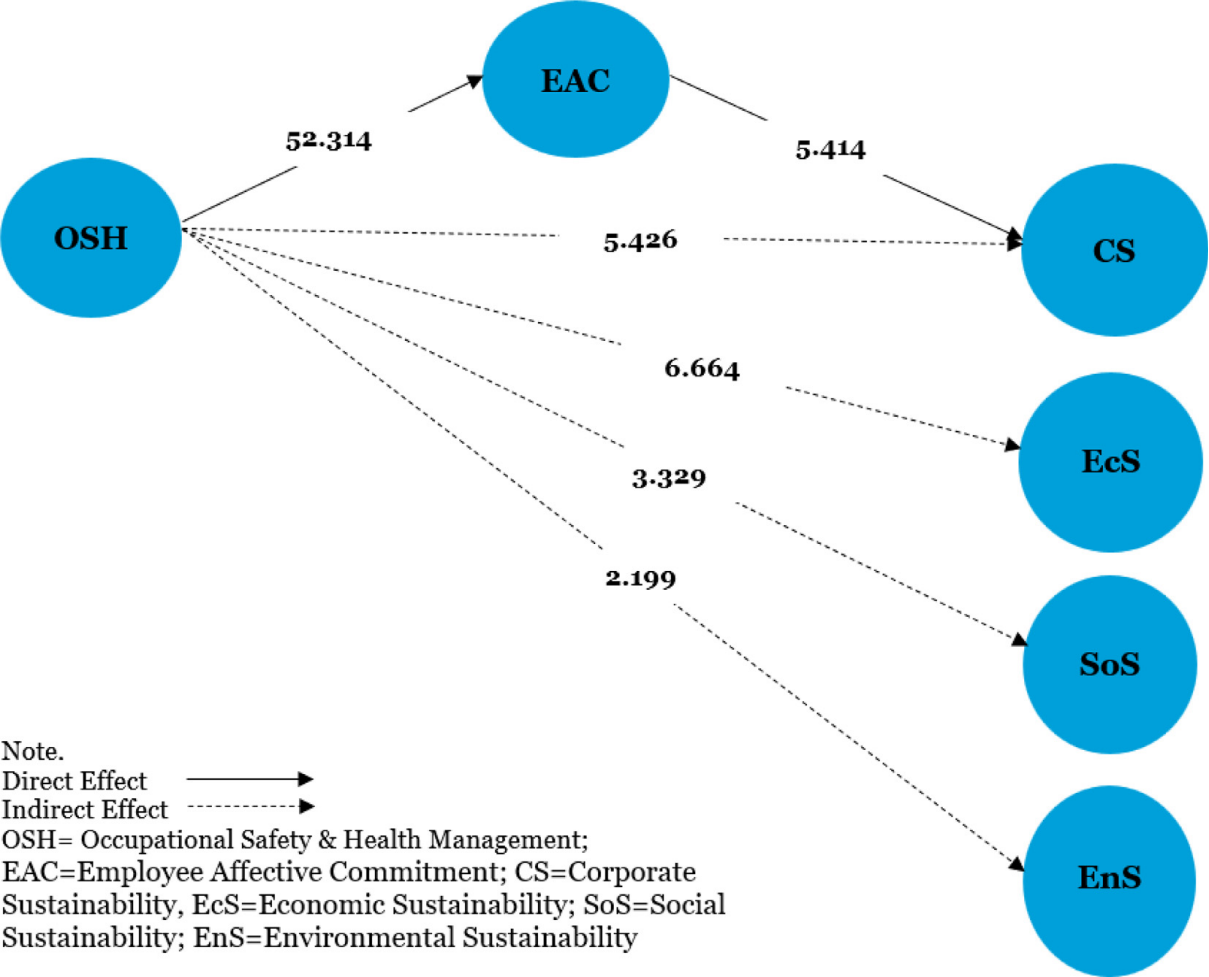
Structural model with indirect paths.

**Fig. 3:**
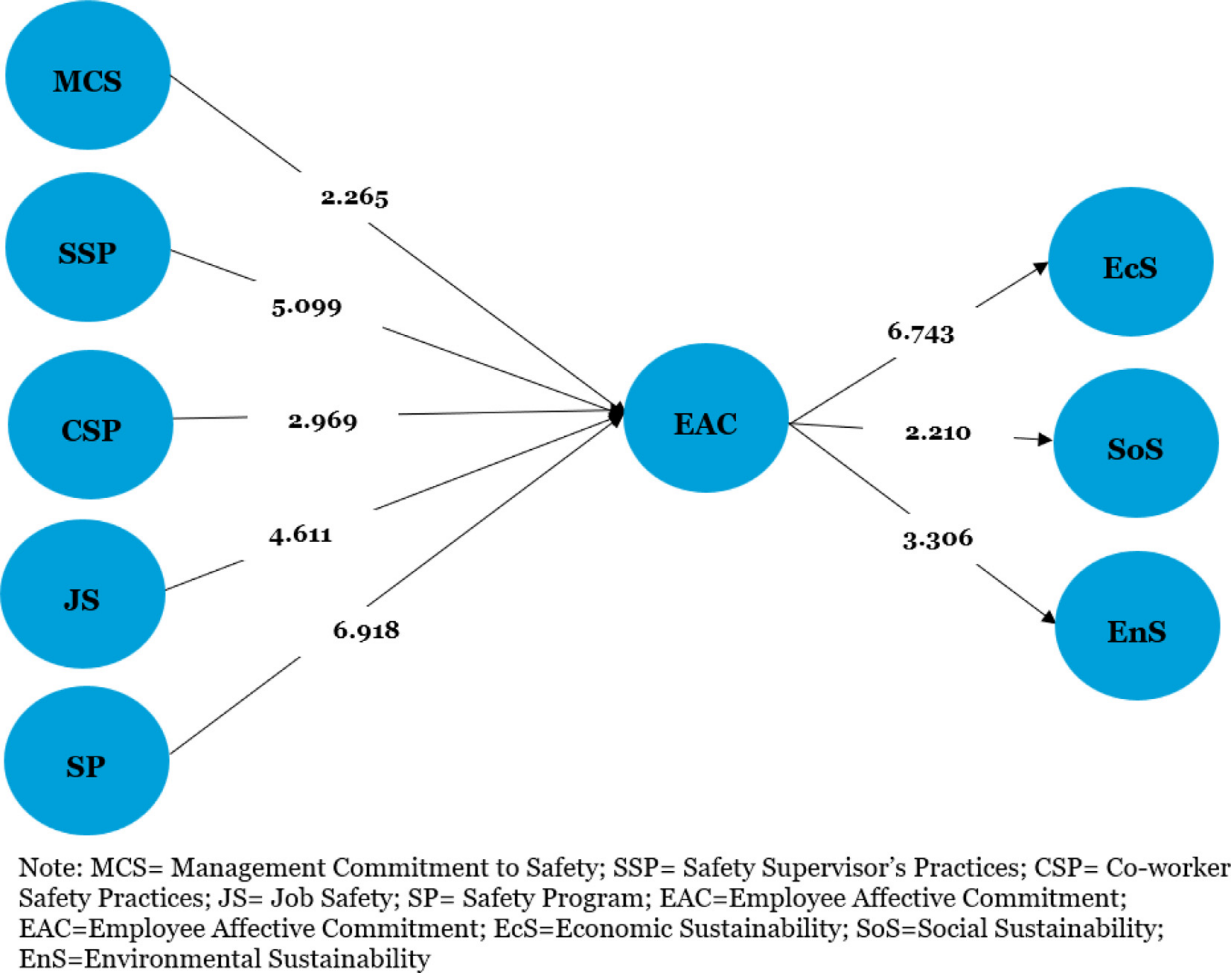
Structural model with direct paths.

Table 4, Fig. 2, Fig. 3 show that OSH (β = 0.842, t = 52.314, p < 0.001) and its dimensions: management commitment to safety (β = 0.097, t = 2.265, p < 0.05), supervisor's safety practices (β = 0.283, t = 5.099, p < 0.001), co-worker safety practices (β = 0.125, t = 2.969, p < 0.05), job safety (β = 0.183, t = 4.611, p < 0.001), and safety program (β = 0.387, t = 6.918, p < 0.001) are all positively related to employee affective commitment, thereby supporting hypotheses H1 and H1a-H1e. Employee affective commitment was also found to be positively related to corporate sustainability (β = 0.316, t = 5.414, p < 0.001), economic sustainability (β = 0.497, t = 6.743, p < 0.001), social sustainability (β = 0.181, t = 2.210, p < 0.05), and environmental sustainability (β = 0.231, t = 3.306, p < 0.001), which supported the H2, H2a, H2b, and H2c hypotheses.

**Table 4:** Structural model results

H	Path	Beta	Standard error	t-Statistics	Results
H1	OSH > EAC	0.847	0.027	31.379∗∗	Supported
H1a	MCS > EAC	0.094	0.058	1.630	Not supported
H1b	SSP > EAC	0.293	0.080	3.644∗∗	Supported
H1c	CSP > EAC	0.122	0.063	1.941∗	Supported
H1d	JS > EAC	0.160	0.059	2.712∗	Supported
H1e	SP > EAC	0.402	0.080	5.033∗∗	Supported
H2	EAC > CS	0.313	0.087	3.581∗∗	Supported
H2a	EAC > EcS	0.497	0.109	4.554∗∗	Supported
H2b	EAC > SoS	0.118	0.041	2.878∗	Supported
H2c	EAC > EnS	0.144	0.045	3.200∗	Supported
H3	OSH > EAC > CS	0.274	0.076	3.604∗∗	Supported
H3a	OSH > EAC > EcS	0.453	0.089	5.093∗∗	Supported
H3b	OSH > EAC > SoS	0.160	0.089	1.802∗	Supported
H3c	OSH > EAC > EnS	0.169	0.088	1.920∗	Supported

Note. CS, Corporate Sustainability; CSP, Co-worker Safety Practices; EAC, Employee Affective Commitment; EcS, Economic Sustainability; EnS, Environmental Sustainability; JS, Job Safety; MCS, Management Commitment to Safety; OSH, Occupational Safety & Health Management; SSP, Safety Supervisor's Practices; SP, Safety Programme; SoS, Social Sustainability; ∗ = significant at 0.05; ∗∗ = significant at 0.001.

Table 4 and Fig. 2 also demonstrate that employee affective commitment significantly mediates the relationship between OSH and corporate sustainability (β = 0.275, t = 5.426, p < 0.001), OSH and economic sustainability (β = 0.418, t = 6.664, p < 0.001), OSH and social sustainability (β = 0.210, t = 3.329, p < 0.001), and OSH and environmental sustainability (β = 0.158, t = 2.199, p < 0.05). Thereby, we can conclude that H3, H3a, H3b, and H3c are well-supported by the data. Succinctly, both OSH and employee affective commitment are crucial constructs that are able to promote greater corporate sustainability through their dimensions. We also tested the direct relationship between OSH and corporate sustainability and its dimensions, which was found to be positively significant. With reference to Zhao et al.’s [57] mediating analysis procedure, it can be concluded that the significant mediating hypotheses are considered partial mediations. Succinctly, both OSH and employee affective commitment are crucial constructs that can promote greater corporate sustainability within their dimensions, except for environmental sustainability.

For the model goodness of fit (GoF), it was tested through several recommended techniques, including computing the standardized root mean square residual by Hu and Bentler [58], Henseler et al. [59], and GoF method by Tenenhaus et al. [60]. Based on the statistical result, it reveals that the standardized root mean square residual common factor model score is less than the threshold value of 0.10, whereas the GoF value with Tenenhaus et al. [60]'s criterion is indicated as 0.495, which is considered large (GoF>0.36). Thus, the model fit is established.

## 4 Discussion

The findings revealed that OSH is positively related to employee affective commitment. This result is consistent with past studies where OSH was found to be positively related to employee commitment [43,61]. Apart from this, our findings also demonstrated that all dimensions of OSH, such as management commitment to safety, supervisor's safety practices, co-worker's safety practices, job safety, and safety program are positively related to employee affective commitment. These findings are supported by past empirical studies [28,[62], [63], [64], [65]]. Employees are more likely to repay their supervisors or co-workers when the latter care for their well-being at work (for example, by providing safety advice or assisting each other in times of crisis) [62,65]. Ahmad [28] also argued that if an organization is able to provide a safe working environment by minimizing the associated workplace risks, the workers will tend to feel grateful towards their organization, which could enhance their commitment to it eventually.

As speculated, employee affective commitment is positively related to corporate sustainability. This finding is corroborated by past studies [17,20,21,66]. Kim et al. [21] argued that employee affective commitment is positively related to economic sustainability. The authors further depicted that employees who possess a high level of commitment tend to have better performance, motivation, and passion, which can improve the organization's performance (i.e., revenue, productivity). Subsequently, it could also enhance the organization's economic sustainability. Further to this, employees with a high level of commitment towards their organization's environmental goals would be more likely to change their attitudes and behaviors to go after the green values as promoted by the organization. This in turn would further enhance the environmental sustainability of an organization. Similarly, Liu et al. [19] argued that employees who are committed to the environmental protection that their organization is concerned about would be more likely to build up the organization's environmental sustainability.

Interestingly, our findings also statistically show that employee affective commitment significantly mediates the relationship between OSH and corporate sustainability. As depicted by Jilcha and Kitaw [22], occupational safety and health management would predict corporate sustainability in conjunction with the key pillars of sustainability. Despotovic et al. [66] contend that the environment (a safe workplace) and society (employees) are key elements in creating competitiveness for an organization and will further lead to corporate sustainability. In view of these results, when employees have a higher level of commitment towards their organization, it serves as a stepping stone to facilitate and strengthen occupational safety and health management, thereby fostering the development of sustainable practices within the organization.

### 4.1 Theoretical implications

The findings of this study have contributed to the existing literature and expanded the body of knowledge, which can be summarized based on two research objectives. First, this research enriches the OSH literature by giving an exhaustive perspective on which dimension of OSH management will be most effective in predicting employee affective commitment. The extant literature scarcely provides insights on the mechanism that explicates how the dimensions of OSH management influence affective commitment. Surprisingly, we discovered that all five dimensions of OSH management have varying degrees of influence on affective commitment. Based on the findings, each of the dimensions for OSH management can be treated differently and have a different effect on predicting the employee's affective commitment. Therefore, this study has advanced theoretical knowledge and provided new insights into OSH research.

Secondly, this study provides new insights into the corporate sustainability literature in relation to the effects of OSH management on corporate sustainability through employee affective commitment. Studies pertaining to the antecedents of corporate sustainability have been developing for decades. Most of them focus on the employee's behavior [[17], [18], [19], [20], [21]] and organizational factors such as culture [67,68], capabilities [69], and size [70]. However, there has been little empirical research on the relationship between perceived occupational safety and health management and corporate sustainability [22,23]. Through this study, the findings successfully concluded that OSH management is also one of the antecedents in predicting corporate sustainability, which means it should not be overlooked by organizations. Thus, when employees perceive that sound OSH management is in place in an organization, they are more likely to commit to it as they feel safe at the workplace, which ultimately reinforces the organization's ability to achieve corporate sustainability. In accordance with the study conducted by Amponsah-Tawiah [23], superior occupational health and safety management is crucial in contributing towards sustainable development. Besides, Jilcha and Kitaw [22] have found a direct relationship between OSH and sustainable development in conjunction with the key pillars of sustainability. Furthermore, according to BBT [26], a safe workplace can boost a worker's positive emotions (such as joy, love, and contentment) at work. Workers' behaviors such as commitment and satisfaction will have developed as a result of their positive emotions. Thus, lead the organization to achieve sustainability in the aspect of economic, social, and environmental. Furthermore, the environment (i.e., a safe workplace) and society (i.e., employees) are considered key elements in increasing the competitiveness of an organization, which would then lead to corporate sustainability [66].

### 4.2 Practical implications

It is undeniable that workplace risk will always exist, even though effective measures have been put in place to mitigate it. Nevertheless, the consequences could be more severe if no measures were taken. Thus, one of the practical implications of this research is to create knowledge and awareness for employers as well as employees on the importance of OSH management at the workplace and potentially enable them to evaluate the impact of such management on the creation of a sustainable organization. This is because sustainable organizations can gain a competitive advantage and more business opportunities, which are some of the primary goals of most organizations. The findings of this study reveal a more holistic and apparent relationship between the five dimensions of OSH measures and their ramifications on employee commitment in an organization. These findings provide useful information to construction organizations, allowing employers to determine which aspects of occupational safety and health they want to focus on in order to create a safer and healthier workplace. Subsequently, by enhancing the employees' positive behavior (i.e., commitment) in the workplace, this could lead to an effective and sustainable development for the organization.

As this research tested different dimensions of OSH management, it can also better guide policymakers to improve the prevailing policies or laws to minimize the accident rates in Malaysia's construction industry. The improvised policies or laws might urge the organizations to be more committed in ensuring workplace safety and health. Meanwhile, these could also promote better transparency and accountability from the organizations. Several effective strategies could be adopted by construction companies. It is suggested that the management of the construction company provide clear, updated, and transparent safety rules and regulations. Sufficient training, education program, and workshops should be offered to employees regularly to ensure they understand and are aware of the safety rules and regulations. Apart from this, management should form a competent and committed safety OSH committee to conduct regular worksite inspections on the safety compliance of the employees. Whereas the assigned supervisors should be well-trained and understand well about safety and health policies so that they know how to deal with safety issues.

### 4.3 Limitations and future research

This research accommodates several limitations and recommendations, some of which are worth pinpointing for future researchers. Firstly, the collected data was solely from the workers who are currently working at the site in the construction industry, which has constrained the generalizability of the results from those workers in other industries. In Malaysia, occupational safety and health management is applicable to most industries, whether private or public. As a result, the findings of this study may not be applicable in generalizing that the workers are employed in industries other than construction. This is due to the fact that different industries might experience and encounter different levels and types of health and safety issues. Thus, future research can be conducted in the context of any industry other than construction in order to obtain a more generalizable result. Secondly, this study is a cross-sectional one, which means it is not able to examine the effects of OSH on employee affective commitment and corporate sustainability over a longer period of time. In the future, a longitudinal study should be conducted to test the same set of variables over a longer period.

### 4.4 Conclusion

This study broadens the existing body of knowledge and advances the understanding of the relationship between OSH, employee affective commitment, and corporate sustainability. It also affirmed that the objectives of this study are well-ascertained. Firstly, the present study contributes to the existing body of knowledge on OSH in the context of the construction industry by confirming that OSH and its dimensions are positively related to employee affective commitment. Secondly, our study also affirmed that affective commitment is positively related to corporate sustainability and its dimensions: economic sustainability, social sustainability, and environmental sustainability. Lastly, our most prominent finding is that employee affective commitment significantly mediates the relationship between OSH and corporate sustainability and its dimensions. Therefore, we concluded that construction companies and relevant regulators should pay more attention to safety and health policies and practices to promote better corporate sustainability in the aspects of economic, social, and environmental through the enhancement of their employees' affective commitment.

## Conflicts of interest

All authors have no conflicts of interest to declare.

## Figures and Tables

**Table:** 

Perceived occupational safety and health management	Management commitment to safety 1. My management provides an adequate safety training program. 2. My management conducts frequent safety inspections. 3. My management provides safe equipment. 4. My management is strict about working safely when work falls behind schedule. 5. My management gives safety personnel the power they need to do their job. 6. After an unsafe event, my management focuses on how to solve problems and improve safety rather than seeking to pin the blame on specific individuals. Supervisor safety practices 1. My supervisor spends time showing me the safest way to do things at work. 2. My supervisor expresses satisfaction when I perform my job safely. 3. My supervisor talks about values and beliefs about the importance of safety. 4. My supervisor makes sure that we receive appropriate rewards for achieving safety targets on the job. 5. My supervisor behaves in a way that displays commitment to a safe workplace. 6. My supervisor keeps workers informed of safety rules. Co-worker safety practices 1. My co-workers ignore safety rules. 2. My co-workers encourage others to be safe. 3. My co-workers take chances with their safety. 4. My co-workers keep the workplace clean. Job safety 1. Site workers could get hurt easily. 2. The work at the site is unsafe. 3. The work at the site is risky. 4. The work at the site is scary. Safety program 1. Safety training programs are useful for my work. 2. The design of the safety training program is good. 3. Safety training programs really work in my workplace. 4. Safety training programs do apply to my workplace. 5. My management provides an adequate safety education program.
Employee organizational commitment	1. I would be very happy to spend the rest of my career with this organization. 2. I enjoy discussing my organization with people outside of it. 3. I really feel as if this organization's problems are my own. 4. I think that I could easily become as attached to another organization as I am to this one. 5. I do not feel like “part of the family” at my organization. 6. I do not feel “emotionally attached” to this organization. 7. This organization has a great deal of personal meaning for me. 8. I do not feel a strong sense of belonging to my organization.
Corporate sustainability	Social sustainability 1. My organization is committed to improving the welfare of the communities in which it operates. 2. My organization actively participates in social and cultural events. 3. My organization plays a role in society that goes beyond mere profit generation. 4. My organization provides fair treatment to employees. 5. My organization provides training and promotion opportunities to its employees. 6. My organization helps to solve social problems. Economic sustainability 1. My organization obtains the greatest possible profits. 2. My organization tries to achieve long-term success. 3. My organization improves its economic performance. 4. My organization ensures its survival and success in the long run. Environmental sustainability 1. My company cares for and protects the environment. 2. My company seeks to know the possible impacts of climate change on its business. 3. My company is recognized for excellence in cleaner production and pollution prevention management. 4. My company carries out specific initiatives to reduce material consumption. 5. My company carries out specific initiatives to reduce water consumption. 6. My company carries out specific initiatives to reduce energy consumption.
